# Laparoscopic vs. Open Repeat Hepatectomy for Recurrent Liver Tumors: A Propensity Score–Matched Study and Meta-Analysis

**DOI:** 10.3389/fonc.2021.646737

**Published:** 2021-04-22

**Authors:** Jia-Feng Chen, Xiu-Tao Fu, Zheng Gao, Ying-Hong Shi, Zheng Tang, Wei-Ren Liu, Xin Zhang, Qiang Gao, Guang-Yu Ding, Kang Song, Xiao-Ying Wang, Jian Zhou, Jia Fan, Zhen-Bin Ding

**Affiliations:** ^1^Department of Liver Surgery & Transplantation, Liver Cancer Institute, Zhongshan Hospital, Fudan University, Shanghai, China; ^2^Key Laboratory for Carcinogenesis and Cancer Invasion, Chinese Ministry of Education, Shanghai, China

**Keywords:** recurrent liver tumors, repeat hepatectomy, laparoscopic surgery, open surgery, meta-analysis

## Abstract

**Background:** It remains unclear whether the short-term benefits of laparoscopic repeat hepatectomy (LRH) accrue to patients with recurrent liver tumors. The present study aimed to report our own center's experience and perform a meta-analysis to evaluate the safety and feasibility of LRH in comparison with open repeat hepatectomy (ORH) for treating recurrent liver tumors.

**Patients and Methods:** A propensity score–matched study was performed including 426 patients receiving LRH or ORH for recurrent hepatocellular carcinoma between January 2017 and December 2018. Surgical outcomes and perioperative inflammation-based markers, including monocyte-to-lymphocyte ratio, neutrophil-to-lymphocyte ratio, platelet-to-lymphocyte ratio, and systemic immune–inflammation index were collected from medical records and analyzed. Additionally, a systematic literature review was performed to identify relevant studies in PubMed, EMBASE, Web of Science, and Cochrane library databases up to October 1, 2020. Information including patient demographics, pathologic characteristics, and short-term outcomes was extracted and analyzed using random- or fixed-effects models.

**Results:** Of 68 LRHs, 57 were matched with an ORH finally. Our study demonstrated that LRH was significantly associated with less intraoperative blood loss (50 vs. 100 mL; *P* < 0.001), lower rate of hepatic inflow occlusion (10.52 vs. 33.3%; *P* = 0.003), and shorter postoperative hospital stay (5 vs. 6 days; *P* = 0.001) after 1:1 propensity score matching. The operation time, rate of blood transfusion, and postoperative complications were similar between the two groups. Moreover, all four inflammation-based markers were significantly lower in LRH group on postoperative day 1. In the meta-analysis, a total of 12 studies comprising 1,315 patients receiving repeat hepatectomy met the selection criteria. Similar to our own study, the meta-analysis showed shorter hospital stay [standard mean difference (SMD) = −0.51, 95% confidence interval (CI) = −0.79 to −0.22, *P* < 0.001], less intraoperative blood loss (SMD = −0.79, 95% CI = −1.11 to −0.47, *P* < 0.001), and lower rate of major postoperative complications [odds ratio (OR) = 0.35, 95% CI = 0.19–0.66, *P* = 0.001] in the LRH group. There was no difference in the field of overall postoperative complication and operation time between LRH and ORH groups.

**Conclusion:** Compared with ORH, LRH results in relatively better surgical outcomes and faster postoperative recovery. It could be considered a feasible and effective option for the treatment of recurrent liver tumors.

## Introduction

Liver tumor is one of the most common malignant tumors and ranks as the fourth leading cause of cancer-related mortality (1). Hepatocellular carcinoma (HCC) is the most common pathological type of liver tumors, especially in the Asia Pacific region (2). Although liver cancer can be treated by curative hepatectomy with other various approaches, the recurrence rate after primary hepatectomy remains high (3). As for the intrahepatic recurrence, the repeat hepatectomy is still considered to be one of the most important potential curative therapies.

A history of abdominal surgery was once considered a contraindication to laparoscopic operation. However, with the advancement and widespread usage of laparoscopic technique and instruments in recent decades, laparoscopic hepatectomy (LH) has been gaining popularity as an alternative to open hepatectomy. In addition, LH for liver tumors, especially for HCC, has been shown to achieve superior short-term outcomes and equivalent oncological prognosis (4). Besides the inherent movement restrictions and disorientation, adhesion and deformity of the liver caused by previous operation disrupt the liver mobilization and make the identification of important vessels and Glissonian pedicles more difficult. Therefore, patients receiving laparoscopic repeat hepatectomy (LRH) suffer from increasing rates of conversion and postoperative complications (5). It is unclear whether or not patients with recurrent liver tumors benefit from LRH.

Enhanced Recovery After Surgery program is a multimodal perioperative care protocol to accelerate recovery by minimizing the physiologic stress of operations (6). The physiologic stress has been linked to changes of organ functions, which could be reflected by inflammation-based markers. The advantage of LRH in recurrent HCC (rHCC) patients has not been fully elucidated, especially for the relationship between postoperative inflammation and short-term outcomes. Therefore, we explored changes of inflammation-based markers after surgery. Moreover, with the same discharge criteria, hospital stay seems to be an important indicator in evaluation of physical rehabilitation. The factors that affect discharge are complex, including body temperature, liver function, pain, diet, patient choice, and so on. The inflammatory response markers can truly reflect the stress state of the patient, and its recovery is an important aspect of physical rehabilitation. Therefore, we also invested the relationship between inflammation-based markers and hospital stay.

To the best of our knowledge, no randomized controlled trials (RCTs) and even limited retrospective studies have been performed to compare the outcomes between LRH and open repeat hepatectomy (ORH). Although a few systematic reviews have been conducted to assess safety and efficiency of LRH for recurrent liver tumors, some high-quality multicenter studies have been published recently and not included in these reviews (7, 8). Herein, the purpose of this study was to carry out a propensity score–based study and a meta-analysis to compare the postoperative outcomes of patients who underwent LRH with those of patients receiving ORH and produce recommendations on the safe and effective practice for recurrent liver tumors.

## Materials and Methods

### The Propensity Score–Matched Study

From January 2017 to December 2018, 729 consecutive patients received curative hepatectomy for recurrent liver tumors at Liver Cancer Institute, Zhongshan Hospital, Shanghai, China. Patients were excluded if they underwent a two-stage procedure, radiofrequency ablation, or other additional operations simultaneously in this study. Of these, 426 patients diagnosed with rHCC pathologically were included in the analysis. The indications for LRH consisted of the following: (1) Child–Pugh grade A or B liver function that recovered to grade A after liver-protective treatment, (2) no clinical signs of major vessel or extrahepatic organs invaded by tumors, (3) absence of gross ascites or severe complications after the previous operation, and (4) no other noteworthy surgical contraindications. The indications for ORH were similar to those for laparoscopic surgery. The final choice of surgical approach was depended on surgeon's preference and experience. The Ethics Committee of Zhongshan Hospital approved the study design (no. B2020-363), and written informed consent was obtained from each patient.

The surgical procedure and surveillance after repeat hepatectomy were similar with those of primary hepatectomy that we described previously (9). All operations were performed by two experienced hepatobiliary surgeons.

### Data Collection

The data collected included baseline, perioperative, and pathologic characteristics from medical records. The following baseline characteristics were obtained: patient demographics, history of previous hepatectomy, the Child–Pugh classification, and preoperative liver function [hepatitis B virus (HBV) infection status, presence of liver cirrhosis]. The approach to previous operation was considered open when patients had received both open hepatectomy and LH previously.

Perioperative characteristics investigated were as follows: conversion rate, duration of surgery, blood transfusion rate, Pringle maneuver requirements, intraoperative blood loss, postoperative morbidity, 90-day mortality, and duration of hospital stay. Postoperative morbidity was classified according to the Clavien–Dindo classification system (10), and the morbidity with grade II or above was recorded in our analysis. Pathologic characteristics consisted of number of tumors, size of maximum tumor, encapsulation of tumors, and location of tumors. Anterolateral hepatectomy was defined as a resection of tumors from segments II, III, IVb, V, or VI, otherwise regarded as posterosuperior hepatectomy.

In addition, we recorded total bilirubin (TB), aspartate transaminase (AST), alanine transaminase (ALT), and prothrombin time (PT) from liver function tests, and lymphocyte, neutrophil, monocyte, and platelet counts from hematological blood tests carried out on preoperative day and postoperative day (POD) 1 and POD 3. The systemic immune–inflammation index (SII) was measured as platelet count × neutrophil count/lymphocyte count, platelet-to-lymphocyte ratio (PLR), neutrophil-to-lymphocyte ratio (NLR), and monocyte-to-lymphocyte ratio (MLR) were also calculated and compared between the two groups.

### Statistical Analysis

Continuous variables were presented as median (range) or mean ± standard deviation (SD), as appropriate for the data distribution. Continuous variables were compared using Mann–Whitney *U-*test (Wilcoxon rank sum test) or Student's *t*-test. Categorical variables were compared using Pearson χ^2^ test or Fisher's exact test, as appropriate. To minimize the influence of potential selection bias, a 1:1 propensity score matching (PSM) was used based on the following eight factors: age, gender, tumor number, maximum tumor size, tumor location, liver cirrhosis, previous hepatectomy approach, and HBV infection status. The choice of these factors was based on their value in the decision to proceed with LRH or ORH and their influence on surgical outcomes. The PSM was performed using nearest neighbor matching with a caliper width of 0.02 according to the recommendations of Lonjon and colleagues (11). X-tile software version 3.6.1 was used to determine the best cutoff values of four inflammation-based markers. The Kaplan–Meier method was used to calculate the hospitalization rate. The log-rank test was used to compare the significance of hospitalization rate between groups. Cases in the LRH group that were converted to ORH were analyzed in the LRH group according to intention-to-treat principles. Two-tailed *P* < 0.05 was considered to be statistically significant. All analyses were performed using SPSS version 25.0, R software version 4.0.2, and GraphPad Prism 8.

## Systematic Review and Meta-Analysis

### Search Strategy and Selection Criteria

A comprehensive and systematic review search in PubMed, EMBASE, Web of Science, and Cochrane Library databases was performed by two researchers (Jiafeng Chen and Xiutao Fu) independently to retrieve all relevant studies published up to October 1, 2020. The MeSH term and synonyms were as follows: “recurrent liver cancer,” “repeat,” “open hepatectomy,” and “laparoscopic hepatectomy.” The references of eligible studies were also reviewed to identify potential relevant articles. The study was registered with the PROSPERO register of systematic review (registration no. CRD 42020219438) and was conducted according to the search strategy based on PRISMA (Preferred Reporting Items for Systematic Reviews and Meta-Analyses) guidelines (12).

Initially, the titles and abstracts of all extracted records were screened by two researchers (Jiafeng Chen and Xiutao Fu) to exclude review articles, letters, editorials, case reports, and other irrelevant studies. Then, the studies deemed potentially eligible were full-text assessed. All included studies in this meta-analysis satisfy the following criteria. The inclusion criteria were as follows: (1) patients were diagnosed with recurrent liver tumors; (2) patients had been treated by LRH or ORH; and (3) data available on the key surgical outcomes in the two respective groups. The exclusion criteria were as follows: (1) records reported in non-English languages; (2) records did not report complete and clear data of surgical outcomes; and (3) records did not fulfill the above inclusion criteria. The discrepancies were resolved by discussion with a third author (Zheng Gao).

### Data Extraction and Quality Assessment

Data were extracted by two reviewers (Jiafeng Chen and Xiutao Fu) independently from the studies as follows: the first author, year of publication, number of patients, and patients' baseline characteristics. Intraoperative characteristics (e.g., operation time, blood loss, blood transfusion rate, use of Pringle maneuver), short-term outcomes, and pathologic characteristics were also recorded. The quality of included studies was evaluated using the Newcastle–Ottawa Quality Assessment Scale (NOS), which contains selection, outcome, and comparability assessment. A minimum of six scores was identified as high-quality study.

### Statistical Analysis

Odds ratio (OR) with 95% confidence interval (CI) was used for analysis of dichotomous variables, and standard mean difference (SMD) with 95% CI was calculated for continuous data. If means and SDs were not provided, they were imputed from medians and ranges by the method of Hozo et al. (13). The heterogeneity was assessed by the *I*^2^ statistics and Cochran's Q test. When *I*^2^ > 50% and *P* < 0.1, a random-effects model was used. Otherwise, a fixed-effect model was applied. With respect to publication bias, it was assessed by observing asymmetry of funnel plots, which was further evaluated by Egger's and Begg's test. Statistical significance was denoted by *P* < 0.05 except where indicated. All *P-*values were two-tailed. All analyses were performed using R software version 4.0.2 and Review Manager version 5.3.

## Results

### Results of Our Retrospective Study

#### Patients' Characteristics

**Figure 1:**
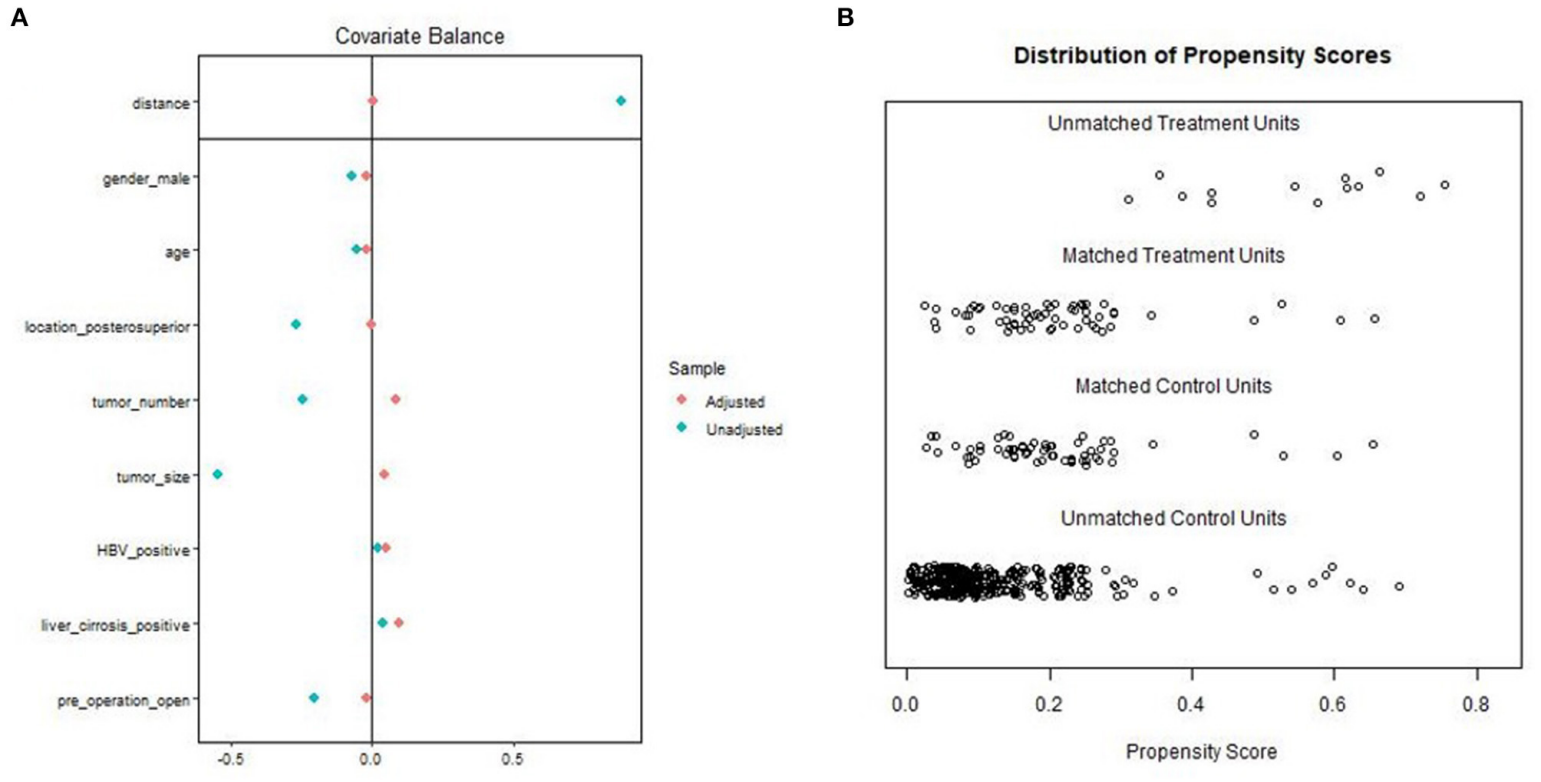
Propensity score matching: **(A)** Dot plot of the standardized mean difference (SMD). **(B)** Propensity score matching jitter plot.

A total of 426 patients underwent repeat hepatectomy for rHCC, 68 treated by LRH and 358 treated by ORH. In the LRH group, six patients required conversion from laparoscopic to open surgery. Of these, three patients had dense intra-abdominal adhesions or the development of portal hypertension and collateral circulation, which may increase the risks of uncontrolled bleeding, injury to important hepatic vessels, biliary trees, and adjacent organs. Another reason of conversion in two patients is failure to localize tumors because of distinct changes of anatomical landmarks. In addition, one patient had conversion to open hepatectomy because of difficulty of dissecting hepatic hilar region. The baseline and pathologic characteristics of the LRH and ORH groups are summarized in Table 1. The ORH group had a larger size of maximum tumor (2 vs. 1.5 cm; *P* < 0.001) and higher rates of posterosuperior resection (50.0 vs. 22.1%; *P* < 0.001). The proportion of previous LH in the LRH group was higher than that in the ORH group (23.5 vs. 5.9%; *P* < 0.001). Owing to the application of 1:1 PSM, 114 patients were selected for comparison, and details of PSM are shown in the dot plot and jitter plot (Figure 1). Baseline characteristics and tumor characteristics were well-balanced between the two groups, with no significant difference (Table 1). In addition, all patients included had ever hepatectomy once or more with liver cirrhosis (stage 4 fibrosis) observed in 62 patients (54.4%). Given these facts, most of our patients underwent partial liver resection in order to reserve enough liver function, while ensuring enough margin (>1 cm). Except for partial resection, five patients received anatomical resection (segmentectomy) in the LRH group and eight in the ORH group (8.7 vs. 14%; *P* = 0.377). In other words, there also was no significant difference in the type of liver resection.

**Table 1:** Patient baseline characteristics and tumor characteristic.

	**Before PSM**			**After PSM**		
**Characteristic**	**LRH (*n* = 68)**	**ORH (*n* = 358)**	***P*-value**	**LRH (*n* = 57)**	**ORH (*n* = 57)**	***P*-value**
Age (years)	56.0 (36.0–78.0)	60.0 (27.0–86.0)	0.099^†^	56.0 (36.0–78.0)	59.0 (34.0–77.0)	0.910^†^
Gender (male/female)	54/14	320/38	0.021^*^	49/8	50/7	0.782^*^
Maximum tumor size (cm)	1.5 (0.6–10.0)	2.0 (0.5–13.0)	<0.001^†^	1.5 (0.6–4.5)	1.7 (0.8–4.5)	0.433^†^
No. of tumors	1.0 (1.0–4.0)	1.0 (1.0–6.0)	0.051^†^	1.0 (1.0–4.0)	1.0 (1.0–2.0)	0.487^†^
Previous surgical approach						
Laparoscopic	16	21	<0.001^*^	7	5	0.542^*^
Open	52	337		50	52	
No. of previous surgery	1.0 (1.0–2.0)	1.0 (1.0–5.0)	0.408^†^	1.0 (1.0–2.0)	1.0 (1.0–3.0)	0.182^†^
Tumor location						
Anterolateral	53	179	<0.001^*^	43	47	0.358^*^
Posterosuperior	15	179		14	10	
HBV (Y/N)	63/5	325/33	0.621^*^	52/5	53/4	1.0^*^
Child–Pugh grade (A/B)	68/0	357/1	1.0^*^	57/0	57/0	1.0^*^
Liver cirrhosis (Y/N)	35/33	180/178	0.857^*^	31/26	31/26	1.0^*^
TB (μmol/L)	11.35 (2.7–37.7)	13.0 (3.1–37.5)	0.120^†^	11.2 (2.7–37.7)	13.2 (4.7–36.4)	0.134^†^
ALT (U/L)	20.5 (6.0–49.0)	21.0 (5.0–219.0)	0.474^†^	20.0 (6.0–43.0)	21.0 (8.0–86.0)	0.512^†^
Albumin (g/L)	45.0 (30.0–53.0)	44.0 (26.0–69.0)	0.742^†^	45.0 (30.0–53.0)	46.0 (36.0–69.0)	0.345^†^
PT (s)	11.6 (10.0–14.0)	11.5 (9.6–15.3)	0.752^†^	11.5 (10.0–14.0)	11.5 (10.2–13.7)	0.986^†^
AFP (ng/mL)						
<20	41	236	0.500^*^	36	36	0.188^*^
20–400	17	84		12	16	
≥400	8	28		7	2	
Tumor capsule						
None and partial	42	202	0.414^*^	33	29	0.452^*^
Complete	26	156		24	28	

*Values are median (range)*.

* *Pearson χ^2^ tests or Fisher's exact test, as appropriate*.

† *Mann–Whitney U-test (Wilcoxon rank sum W-test)*.

*PSM, propensity score matching analysis; LRH, laparoscopic repeat hepatectomy; ORH, open repeat hepatectomy; HBV, hepatitis B virus; TB, total bilirubin; ALT, alanine transaminase; PT, prothrombin time; AFP, α-fetoprotein; Y, yes; N, no*.

#### Comparison of Surgical Outcomes Between LRH and ORH for rHCC

Propensity score–adjusted analyses demonstrated that the median blood loss was significantly lower in the LRH group (50 mL; range = 10–600 mL) than ORH group (100 mL; range = 20–800 mL) (*P* < 0.001). In addition, LRH was associated with less appliance of Pringle maneuver (10.5 vs. 33.3%; *P* = 0.003) and shorter postoperative hospital stay (5 vs. 6 days; *P* = 0.001). The median operation time was similar in the LRH group (131 min; range = 45–415 min) and ORH group (124 min; range = 57–264 min) (*P* = 0.285). With respect to postoperative complications, one patient in LRH group and two in the ORH group developed complications of grade II or above. All three patients experienced pleural effusion requiring drainage, and one patient in the ORH group experienced peritoneal effusion simultaneously. No postoperative mortality occurred in either group (Table 2).

**Table 2:** Surgical outcomes after PSM.

**Outcomes**	**LRH (*n* = 57)**	**ORH (*n* = 57)**	***P*-value**
Conversion	6 (10.5%)	NA	NA
Operation time (min)	131.0 (45.0–415.0)	124.0 (57.0–264.0)	0.285^†^
Blood loss (mL)	50.0 (10.0–600.0)	100.0 (20.0–800.0)	<0.001^†^
Transfusion (yes/no)	1/56	0/57	1.0^*^
Pringle maneuver (yes/no)	6/51	19/38	0.003^*^
Complication (yes/no)	1/56	2/55	1.0^*^
AST (U/L)	103.0 (34.0–2,209.0)	214.0 (77.0–1,916.0)	<0.001^†^
ALT (U/L)	104.0 (19.0–1,828.0)	187.0 (51.0–1,804.0)	<0.001^†^
TB (μmol/L)	25.6 (12.6–75.7)	28.3 (15.2–62.4)	0.069^†^
PT (s)	12.9 (11.1–17.3)	13.7 (11.5–17.2)	<0.001^†^
Hospital stay (days)	5.0 (3.0–13.0)	6.0 (4.0–33.0)	0.001^†^

*Values are median (range)*.

* *Pearson χ^2^ tests or Fisher's exact test, as appropriate*.

† *Mann–Whitney U-test (Wilcoxon rank sum W-test)*.

*LRH, laparoscopic repeat hepatectomy; ORH, open repeat hepatectomy; AST, aspartate transaminase; ALT, alanine transaminase; TB, total bilirubin; PT, prothrombin time*.

**Figure 2:**
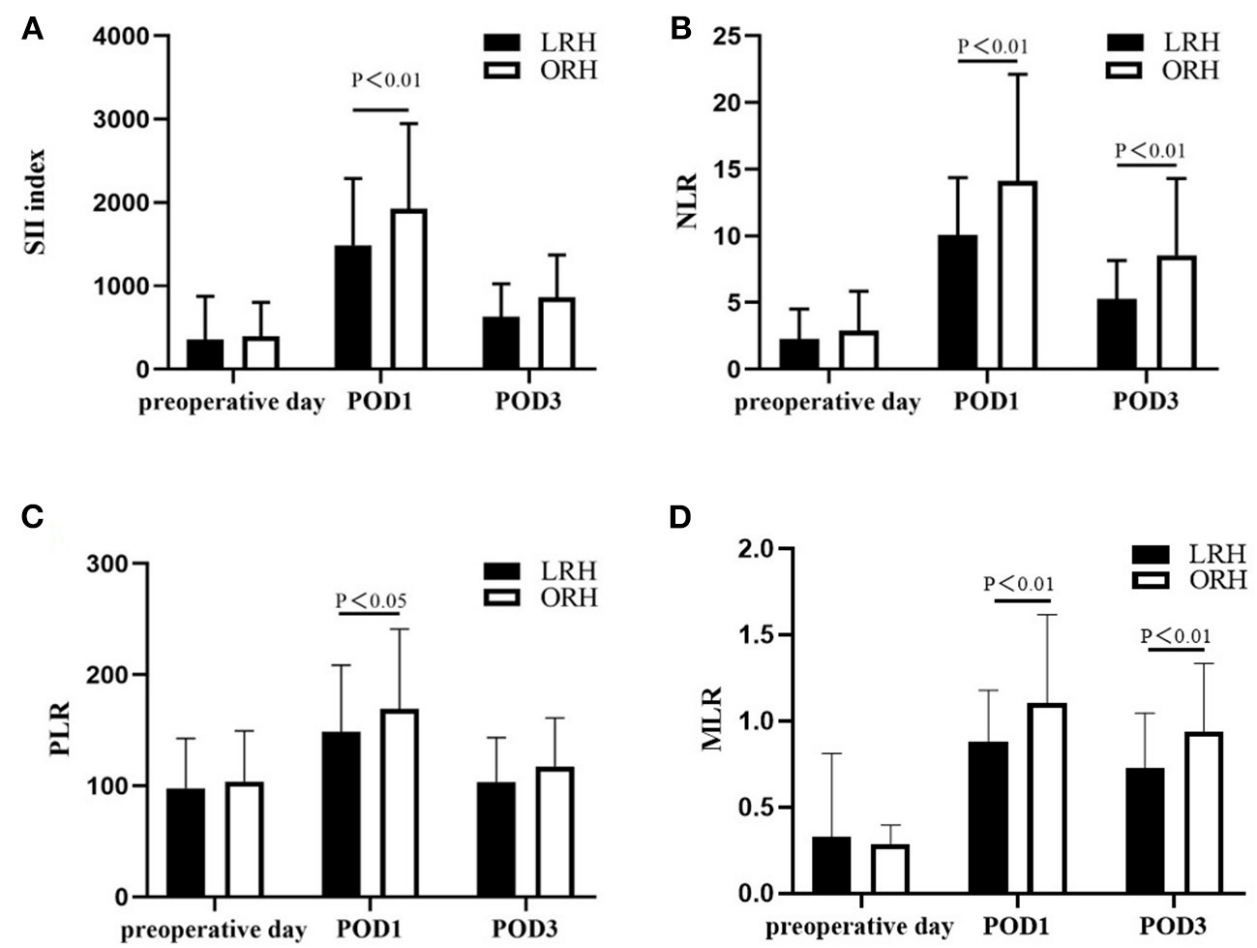
Changes in the level of **(A)** SII, **(B)** NLR, **(C)** PLR, and **(D)** MLR on preoperative day, postoperative day (POD) 1 and POD 3. Values are presented as mean ± standard deviation. SII, systemic immune–inflammation index; NLR, neutrophil-to-lymphocyte ratio; PLR, platelets-to-lymphocyte ratio; MLR, monocyte-to-lymphocyte ratio.

**Figure 3:**
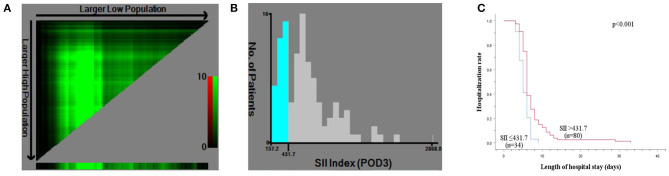
X-tile analysis for calculating the cutoff point of SII on postoperative day (POD) 3. **(A)** X-tile plot of SII on POD 3. **(B)** The optimal cutoff point shown on a histogram of entire cohort. **(C)** Kaplan–Meier plot of association between SII ≤ 431.7 and hospitalization rate.

The levels of ALT, AST, TB, and PT, especially on the peak day, were lower in the LRH group than those in the ORH group (*P* < 0.001, *P* < 0.001, *P* = 0.069, and *P* < 0.001, respectively) (Table 2). In addition, the mean values of SII, NLR, PLR, and MLR on POD 1 and POD 3 are summarized in Figure 2. The four inflammation-based markers were comparable in the two groups before surgery. As compared with those of LRH group, SII, NLR, PLR, and MLR in the ORH group were significantly higher on POD 1 [1,929.7 ± 1,017.3 vs. 1,490.0 ± 797.0 (*P* < 0.001); 14.1 ± 8.0 vs. 10.1 ± 4.3 (*P* < 0.001); 169.1 ± 71.9 vs. 148.6 ± 60.0 (*P* = 0.037); 1.11 ± 0.51 vs. 0.88 ± 0.30 (*P* = 0.001), respectively]. Although all these four markers were elevated in the ORH group on POD 3, only NLR and MLR were significantly higher than those in the LRH group [8.5 ± 5.8 vs. 5.3 ± 2.9 (*P* < 0.001); 0.94 ± 0.40 vs. 0.73 ± 0.32 (*P* = 0.003)]. We also invested the relationship between inflammation-based markers and hospital stay by performing a quantitative X-tile software analysis. The optimal value was produced when applying 431.7 of SII on POD 3 as cutoff value to divide the cohort into two subsets (Figures 3A,B). The Kaplan–Meier plot showed that SII ≤ 431.7 on POD 3 was associated with shorter hospital stay (*P* < 0.001) (Figure 3C). These results provide evidence that LRH was associated with faster postoperative recovery.

## Results of the Meta-Analysis

### Study Characteristics and Quality Assessment

**Figure 4:**
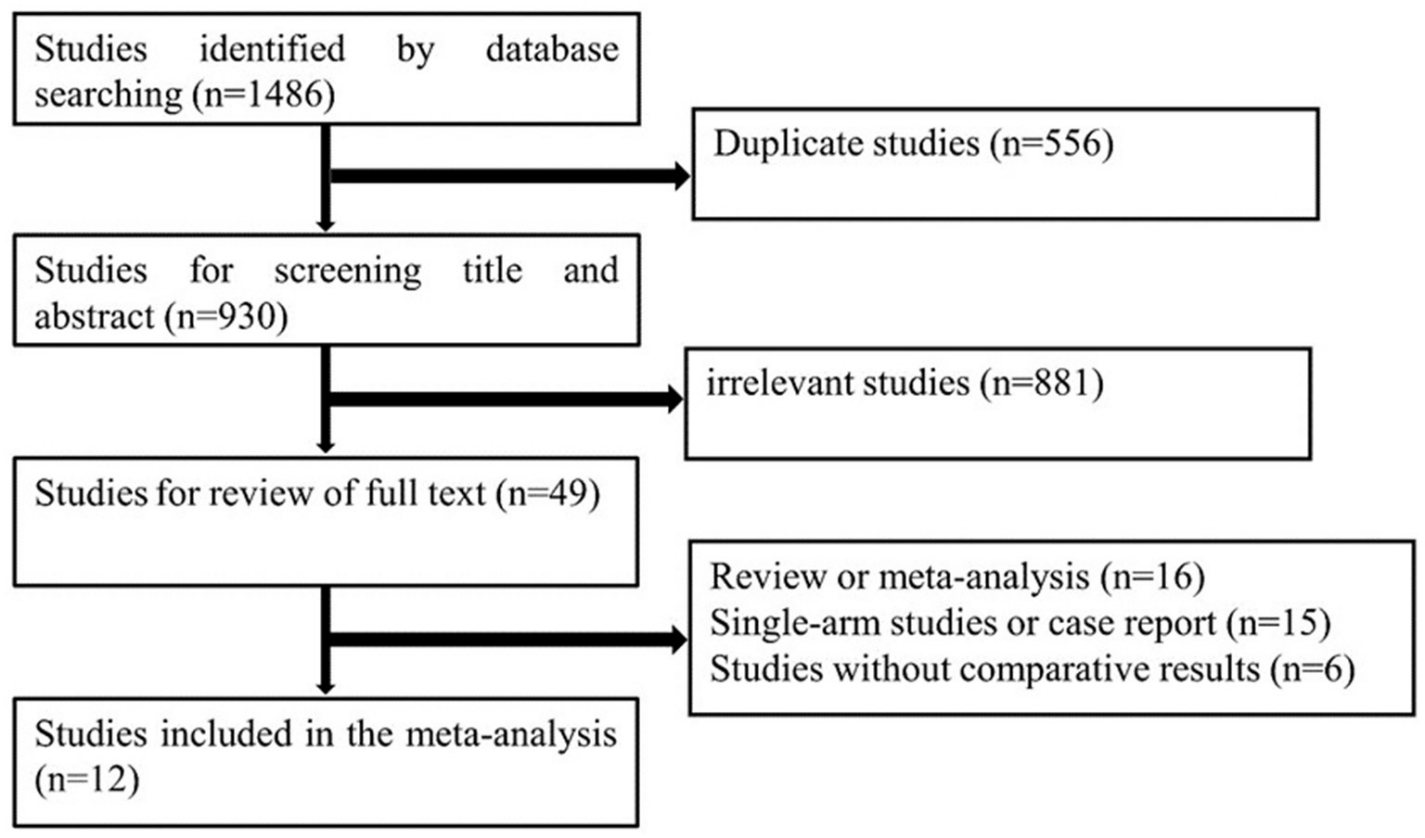
Flow diagram of the selecting process.

The search strategy identified a total of 1,486 citations from the electronic databases. After removing duplicates and studies that did not fulfill the eligibility criteria, full-text review occurred for 56 studies. Of these, 12 studies (14–24) compared LRH with ORH for 1,315 patients diagnosed with recurrent liver tumors and provided complete data on patients' characteristics and surgical outcomes. There were 602 and 713 patients in the LRH and ORH, respectively. A flow diagram of the selection process was outlined in Figure 4. The characteristics of eligible studies are summarized in Table 3. Of the studies included, five were conducted in Japan, three in China, one in Europe, one in Singapore, one in France, and one in 42 liver surgery centers around the world. A summary of NOS scores of all studies is given in Table 4. Scores of all studies ranged from 7 to 8, which were assessed as high quality.

**Table 3:** Summary of characteristics of included studies.

**References**	**Study design**	**Country**	**Group**	**No**.	**Gender (M/F)**	**Age (years)**	**Child–Pugh grade (A/B)**	**Liver cirrhosis (yes/no)**	**Previous operation (OH/LH)**	**Tumor size (cm)**	**Pathology**
Kanazawa et al. (14)	RM	Japan	Lap	20	15/5	70 (46–83)	19/1	7/13	15/5	1.7 (0.7–3.5)	HCC = 20
			Open	20	19/1	65 (43–74)	17/3	7/13	NA	2.2 (1.3–4.1)	HCC = 20
Chan et al. (15)	RM	China	Lap	11	8/3	61 (43–80)	11/0	8/3	6/5	2.0 (1.0–4.5)	HCC = 11
			Open	22	16/6	62 (43–76)	NA	NA	NA	2.0 (1.0–5,0)	HCC = 22
Zhang et al. (16)	P	China	Lap	31	26/5	54 (37–66)	NA	NA	31/0	2.5 ± 1.0	HCC = 31
			Open	33	27/6	59.5 (34–65)	NA	NA	33/0	3.8 ± 1.1	HCC = 33
Hallet et al. (18)	PSM	France	Lap	27	20/7	63.6 (59–70.9)	NA	NA	NA	NA	CRLM = 27
			Open	81	50/31	62.8 (57.5–70.3)	NA	NA	NA	NA	CRLM = 81
Liu et al. (17)	PSM	China	Lap	30	23/7	56.5 (27–79)	30/0	26/4	21/9	2.1 (1.0–5.0)	HCC = 30
			Open	30	28/2	48.5 (28–79)	27/3	26/4	NA	2.45 (1.0–4.3)	HCC = 30
Noda et al. (19)	R	Japan	Lap	20	15/5	68.8 ± 9.7	19/1	8/12	12/8	2.41 ± 1.26	HCC = 15/CRLM = 5
			Open	48	39/9	67.2 ± 8.4	44/4	16/32	46/2	2.21 ± 1.09	HCC = 36/CRLM = 12
Ome et al. (20)	R	Japan	Lap	33	26/7	73 (45–84)	33/0	13/20	21/12	1.80 (0.4–4.5)	HCC = 16/M = 15/B = 2
			Open	37	27/10	71 (45–84)	36/1	10/27	34/3	2.40 (0.7–5.5)	HCC = 16/M = 16/B = 2/CCC = 1/others = 2
Goh et al. (21)	PSM	Singapore	Lap	20	18/2	68.5 (67–71.75)	NA	7/13	7/13	2.00 (1.15–2.775)	HCC = 20
			Open	20	18/2	69 (63–72.25)	NA	7/13	NA	2.60 (1.50–3.0)	HCC = 20
Inoue et al. (22)	PSM	Japan	Lap	37	25/12	69 (45–86)	37/0	NA	NA	2.2 (0.8–5.2)	HCC/CCC = 18/others = 19
			Open	37	23/14	69 (42–81)	37/0	NA	NA	2.2 (0.5–4.3)	HCC/CCC = 19/others = 18
van der Poel et al. (23)	PSM	7 European countries	Lap	105	62/43	61 ± 10.7	NA	NA	66/39	2.8 (1.9–4.4)	CRLM = 105
			Open	105	62/43	62 ± 9.6	NA	NA	69/36	3.0 (2.0–4.0)	CRLM = 105
Onoe et al. (24)	R	Japan	Lap	30	23/7	70.9 (50–85)	30/0	6/24	21/9	1.25 (0.08–3.5)	HCC = 30
			Open	42	30/12	72.0 (59–88)	34/8	16/26	36/6	1.75 (0.5–6.0)	HCC = 42
Morise et al. (25)	PSM	42 liver surgery centers	Lap	238	181/57	67.1 ± 11.8	NA	177/61	181/57	2.75 ± 2.88	HCC = 238
			Open	238	184/54	66.4 ± 10.2	NA	174/64	187/51	2.77 ± 2.64	HCC = 238

*OH, open hepatectomy; LH, laparoscopic hepatectomy; M, male; F, female; RM, retrospective matched cohort; Lap, laparoscopic; HCC, hepatocellular carcinoma; NA, not available; P, prospective cohort; PSM, propensity score–matched cohort; R, retrospective cohort; CRLM, colorectal liver metastasis; CCC, central cholangiocarcinoma; B, combined HCC and CCC*.

**Table 4:** Quality assessment using Newcastle–Ottawa Scale (NOS).

**References**	**Selection (out of 4)**				**Comparability (out of 2)**	**Outcomes (out of 3)**			**NOS score**
	**Representativeness of exposed cohort**	**Selection of non-exposed cohort**	**Exposure**	**Outcome of interest not present at start**		**Assessment of outcome**	**Follow-up**	**Adequacy of follow-up**	
Kanazawa et al. (14)	*	*	*	*	**	*	Unclear	Unclear	7
Chan et al. (15)	*	*	*	*	**	*	Unclear	Unclear	7
Zhang et al. (16)	*	*	*	*	**	*	*	Unclear	8
Hallet et al. (18)	*	*	*	*	**	*	*	Unclear	8
Liu et al. (17)	*	*	*	*	**	*	*	Unclear	8
Noda et al. (19)	*	*	*	*	**	*	Unclear	Unclear	7
Ome et al. (20)	*	*	*	*	**	*	Unclear	Unclear	7
Goh et al. (21)	*	*	*	*	**	*	*	Unclear	8
Inoue et al. (22)	*	*	*	*	**	*	Unclear	Unclear	7
van der Poel et al. (23)	*	*	*	*	**	*	Unclear	Unclear	7
Onoe et al. (24)	*	*	*	*	**	*	Unclear	Unclear	7
Morise et al. (25)	*	*	*	*	**	*	*	Unclear	8

### Surgical Outcomes of LRH vs. ORH

**Figure 5:**
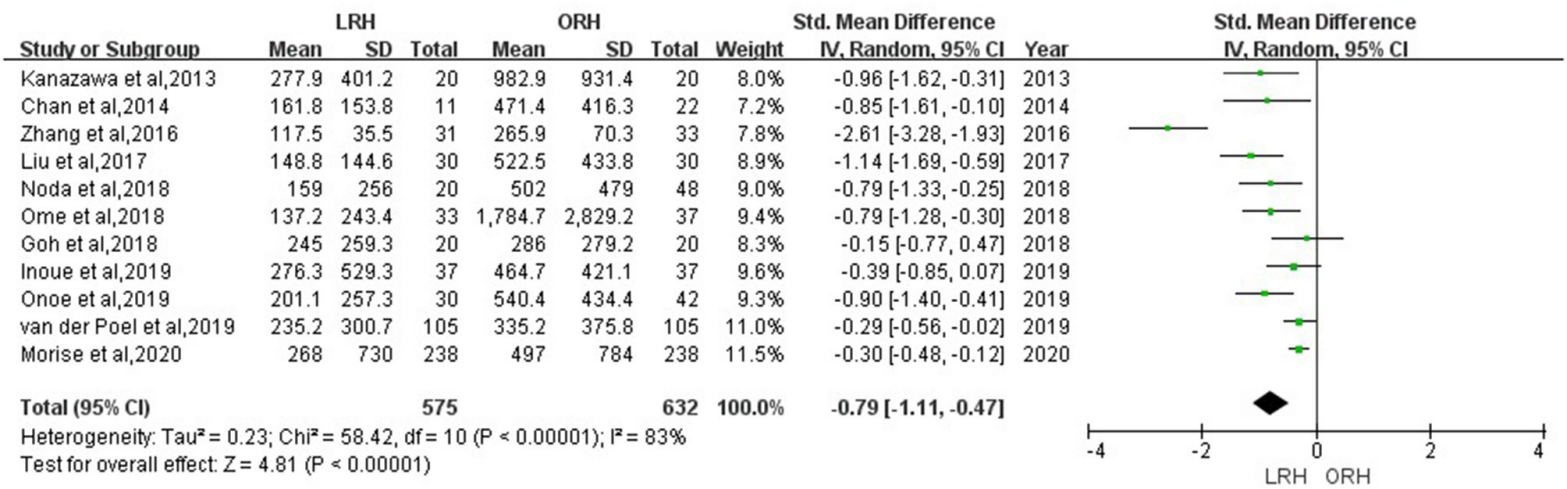
Forest plots comparing blood loss between LRH group and ORH group.

**Figure 6:**
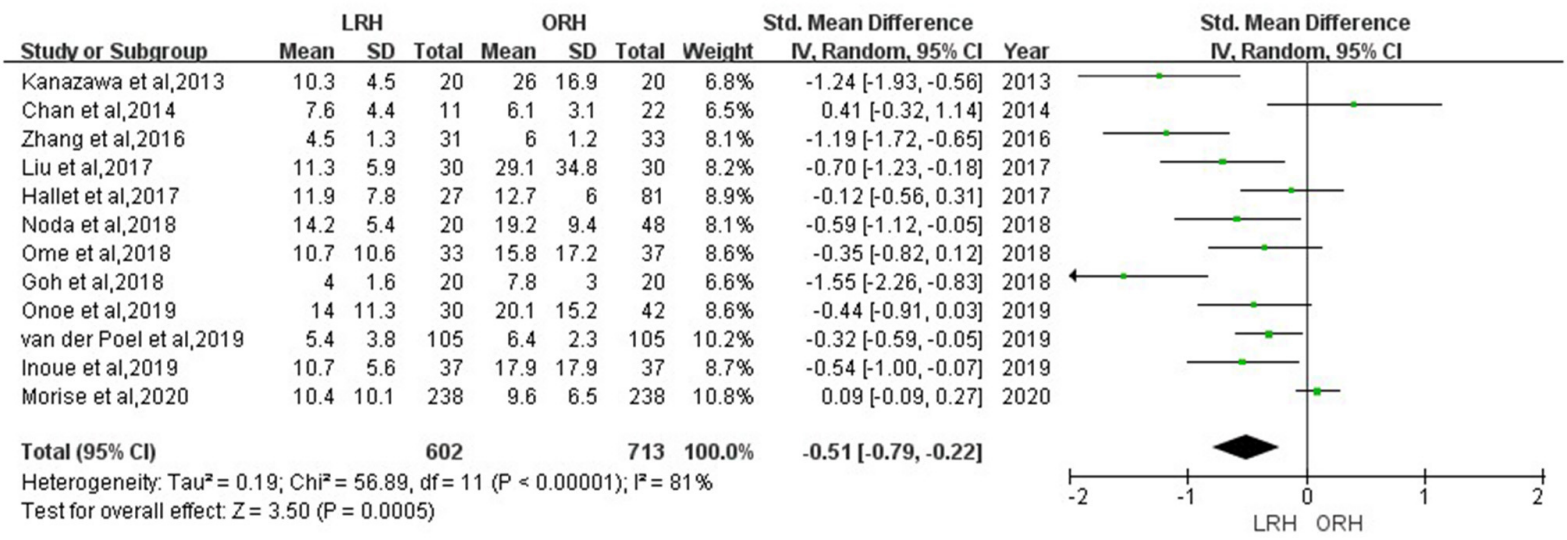
Forest plots comparing hospital stay between LRH group and ORH group.

**Figure 7:**
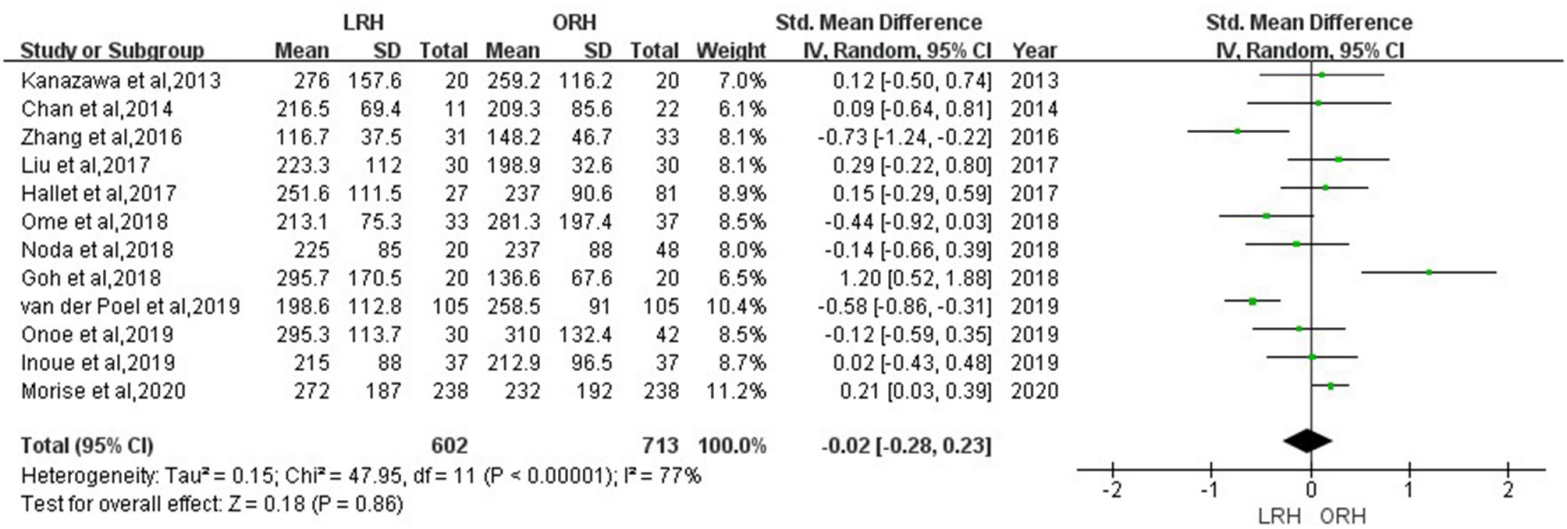
Forest plots comparing operation time between LRH group and ORH group.

**Figure 8:**
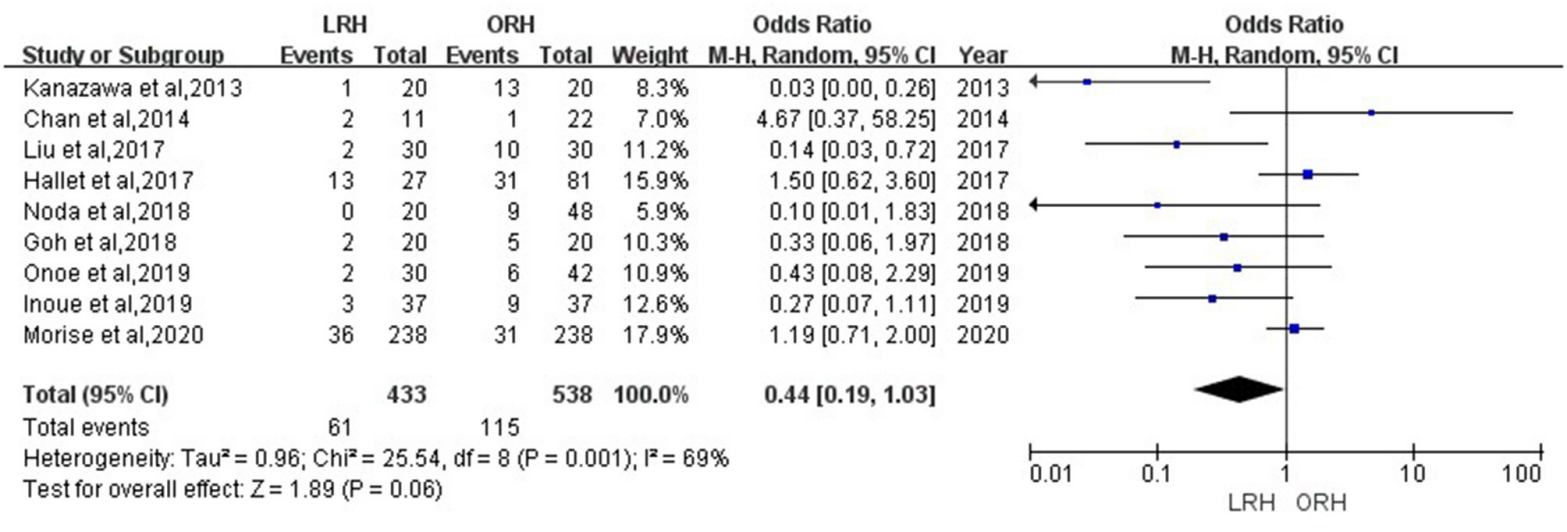
Forest plots comparing overall postoperative complications rate between LRH group and ORH group.

**Figure 9:**
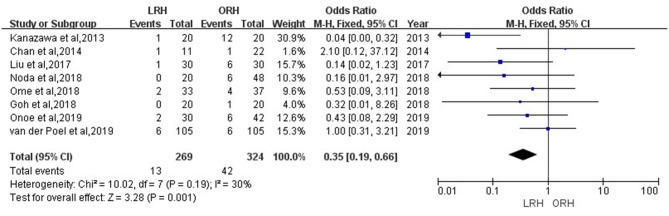
Forest plots comparing major postoperative complications rate between LRH group and ORH group.

**Figure 10:**
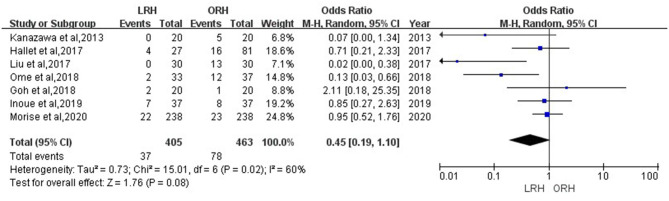
Forest plots comparing blood transfusion rate between LRH group and ORH group.

**Figure 11:**
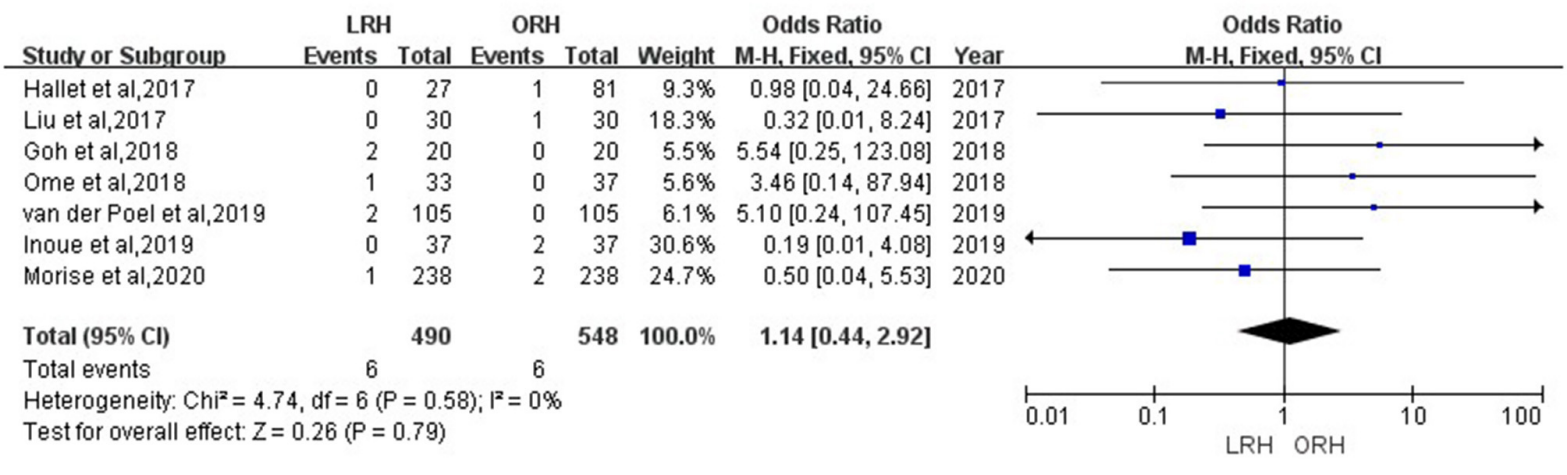
Forest plots comparing mortality between LRH group and ORH group.

According to this meta-analysis, the intraoperative blood loss was significantly lower in the LRH than that in the ORH group (SMD = −0.79, 95% CI = −1.11 to −0.47, *P* < 0.001) (Figure 5). All these 12 studies had reported duration of surgery and postoperative hospital stay. The pooled data indicated that duration of hospital stay was reduced in the LRH group in comparison with that in the ORH group (SMD = −0.51, 95% CI = −0.79 to −0.22, *P* < 0.001) (Figure 6). However, the operation time did not differ significantly between the two groups (SMD = −0.02, 95% CI = −0.28 to 0.23, *P* = 0.86) (Figure 7). Furthermore, nine studies had provided data of postoperative complications, with eight providing major complications. Overall complication rate did not differ significantly between the two groups (OR = 0.44, 95% CI = 0.19–1.03, *P* = 0.06) (Figure 8), whereas the major complications were significantly decreased in LRH group when compared to ORH group (OR = 0.35, 95% CI = 0.19–0.66, *P* = 0.001) (Figure 9). In addition, there were no significant differences in terms of transfusion rate (OR = 0.45, 95% CI = 0.19–1.10, *P* = 0.08) (Figure 10) and mortality (OR = 1.14, 95% CI = 0.44–2.92, *P* = 0.79) (Figure 11).

### Publication Bias

The publication bias evaluation for the meta-analysis of operation time is shown in Supplementary Figure 1. There was no obvious asymmetry in the funnel plot. In addition, the Begg test (*P* = 0.681) and Egger test (*P* = 0.942) further showed that there was no potential publication bias among studies.

## Discussion

To explore the advantages of LRH over ORH in treating recurrent liver tumors, we performed the present PSM analysis to minimize the selection bias and then compared surgical outcomes between the two groups. The results indicated that LRH had obvious advantages, such as less intraoperative blood loss and use of Pringle maneuver. The reasons for reduced blood loss and use of Pringle maneuver were mainly the positive pressure of pneumoperitoneum and magnified view of laparoscopic approach. Moreover, patients undergoing LRH seems to have faster postoperative recovery because LRH was associated with better postoperative liver function and shorter hospital stay. Although the exact mechanism of enhanced recovery after LRH has not been elucidated clearly, we presumed that attenuation of postoperative inflammation might play an important role as the inflammation-based markers were significantly lower in LRH group. Pringle maneuver was more applied in ORH group, which may cause ischemia–reperfusion injury and postoperative liver dysfunction (26). Thus, how to minimize ischemia–reperfusion and maximize the protection of liver function should be one of the focuses in the surgery. We also demonstrated that SII ≤ 431.7 on POD 3 was associated with shorter hospital stay, which indicates this index may be practical in predicting the faster postoperative recovery. Interestingly, similar with published study, most of matched patients in our retrospective cohort were diagnosed with rHCC accompanied with liver cirrhosis, indicating that LRH can be a safe and efficient procedure for cirrhotic patients (27, 28). However, the operation time, blood transfusion rate, and incidence of postoperative complications in the LRH group were similar to those in the ORH group.

The first reported PSM analysis of LRH vs. ORH for rHCC suggested that there was significant difference in postoperative outcomes between two approaches, including lower morbidity rate, reduced blood loss, and shorter hospital stay in the LRH group (17). Contrary to that, a similar study comparing LRH and ORH for colorectal liver metastases failed to show difference of surgical outcomes except surgery-specific morbidity rate (18). The contradiction may derive from the difference in the baseline characteristics between these two diseases and various surgical skills and techniques, as well as the selection bias caused by the retrospective study design. Furthermore, those analyses did not include many possible remaining confounders into the PSM model, such as the location of tumors and approach to previous operation, which may influence the odds of conversion and other surgical outcomes. As previously reported, there were more severe adhesions if the previous hepatectomy was performed by open approach (29). Thus, the present PSM analysis built a model based on eight variables, including age, gender, tumor number, maximum tumor size, tumor location, liver cirrhosis, HBV infection status, and previous hepatectomy approach. After balancing the baseline characteristics using PSM, there was no apparent difference in postoperative morbidity between LRH and ORH.

In one recent meta-analysis performed by Liang et al., the multicenter propensity score–based analysis conducted by Morise et al. was not included, which comprises 476 matched patients (8, 25). To make the meta-analysis more convincing, we combined the results of the study above with those from 11 previous studies. Our analysis included 1,315 patients in total, which was almost twice the patients of the most recent meta-analysis. With the exception of major postoperative complications, the result of meta-analysis was comparable to the present propensity score–based study. As compared with ORH, LRH was associated with less blood loss and faster postoperative recovery with equivalent morbidity rate. The smaller wound and lower postoperative pain help patients walk sooner after operation, which then result in shorter hospital stay and enhanced recovery. These data have provided a more comprehensive conclusion regarding the safety and efficiency of LRH for the treatment of recurrent liver tumors.

As LRH presents more challenges because of intra-abdominal adhesions, especially in patients with severe portal hypertension, LH was considered a contraindication for recurrent liver tumors. Besides, Belli et al. reported that the selected patients for LRH should satisfy the following criteria: well-preserved liver function without signs of severe portal hypertension, a maximum size of 5 cm, and tumor located in anterolateral segments (30). However, with the improvement of laparoscopic surgical techniques and instruments, we also carried out LRH for rHCC located in posterosuperior segments or rHCC with maximum size of >5 cm. It has been reported that LH could reduce formation of adhesions and damage to liver parenchyma, collateral vessels, and surrounding structures (31, 32). The pneumoperitoneum and magnified view of laparoscopic approach make the adhesiolysis more meticulous, contributing to less blood loss. In addition, LH was suggested for patients with poor liver function because of the advantages in surgical outcomes, including smaller incision and less hepatic mobilization and blood loss (25). Notwithstanding these advantages, the Southampton guidelines stated that LRH should be performed by experienced surgeons and avoided in the early phase of learning curve (33). Besides, the proper trocar placement should be adjusted according to operation custom of the surgeon, as well as the changed liver anatomy and formed adhesions caused by previous hepatectomy. Moreover, for the consideration of future abdominal operations, it is better to avoid unnecessary extensive adhesiolysis when the adhesion does not affect the operative procedure (34, 35).

Although our study combined a PSM analysis with a meta-analysis in order to draw a more definitive conclusion, several limitations of this study must be considered. First, there are still selection biases in our own data as this study is a retrospective analysis of a single center. Despite the PSM analysis, the level of evidence still cannot compete with that of RCT because PSM cannot control for other potential confounders we do not include. Second, the included patients in our center are still under follow-up, and the data of long-term outcomes are not complete and adequate in our own study, as well as other published studies. Thus, we did not evaluate long-term oncologic outcomes in the present PSM analysis and meta-analysis. Third, most of the included studies in the meta-analysis were retrospective case series in a single center without proper patient randomization, which may be inclined to cause selection bias. Significant heterogeneity was found in some outcomes between the included studies, which may be attributed to study designs, characteristics of the patients, various surgical equipment and procedure, and different indications for LRH with recurrent liver tumors. In view of these limitations, studies with larger scale and RCTs with short- or long-term oncological outcomes should be carried out to further confirm the advantages of LRH.

## Conclusions

We compared the perioperative outcomes of LRH and ORH for patients with recurrent liver tumors. Although there are several challenges mentioned previously, LRH can be an appropriate minimally invasive procedure to treat recurrent liver tumors for selected patients because it presents a similar risk of postoperative complications and a faster postoperative recovery. Nonetheless, standard procedure of LRH should be established, and further large-scale studies are required to determine specific indications of LRH for recurrent liver tumors.

## Data Availability Statement

The raw data supporting the conclusions of this article will be made available by the authors, without undue reservation.

## Ethics Statement

The studies involving human participants were reviewed and approved by Ethics Committee of Zhongshan Hospital of Fudan University. The patients/participants provided their written informed consent to participate in this study.

## Author Contributions

J-FC, X-YW, JF, and Z-BD conceived and designed the study. J-FC, X-TF, ZG, Y-HS, ZT, W-RL, XZ, KS, QG, and G-YD participated in the database search, data collection, and quality assessment. J-FC, X-TF, ZG, Y-HS, ZT, W-RL, XZ, QG, and JZ performed the statistical analysis. J-FC, X-TF, and ZG edited the paper. All authors provided critical revision of article and final approval of article.

## Conflict of Interest

The authors declare that the research was conducted in the absence of any commercial or financial relationships that could be construed as a potential conflict of interest.

## Supplementary Material

Supplementary Figure 1 Funnel plot of publication bias based on studies reporting operation time.
