# Supplementary figures and images for: Laparoscopic vs. Open Repeat Hepatectomy for Recurrent Liver Tumors: A Propensity Score–Matched Study and Meta-Analysis
Source: Front Oncol. 2021 Apr 22;11:646737. doi: 10.3389/fonc.2021.646737 (PMC8100033; doi:10.3389/fonc.2021.646737)

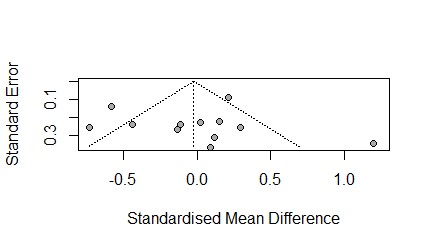

Supplement: Supplementary Figure 1 — Funnel plot of publication bias based on studies reporting operation time. [file Image_1.JPEG]
